# Endoscopic ultrasonography-guided vascular intervention for bile duct–jejunal anastomotic variceal bleeding

**DOI:** 10.1055/a-2582-4026

**Published:** 2025-04-29

**Authors:** Takashi Kondo, Kazuo Hara, Nozomi Okuno, Shin Haba, Takamichi Kuwahara, Minako Urata, Yoshitaro Yamamoto

**Affiliations:** 1 538363 Gastroenterology, Aichi Cancer Center Hospital, Nagoya, Japan

Extrahepatic portal venous obstruction rarely causes ectopic varices in the duodenum or small intestine. Ectopic varices formed by extrahepatic portal venous obstruction can cause bleeding, which is difficult to treat 1 , but there have been several case reports of successful hemostasis using endoscopic ultrasonography (EUS)-guided vascular intervention 2 3 4 . In this study, we report a case in which bile duct–jejunal anastomotic variceal bleeding was successfully stopped using an EUS-guided vascular intervention.

The patient was a 79-year-old man who had undergone pancreaticoduodenectomy and combined portal vein resection for pancreatic cancer. He presented with dark red stool, and blood sampling revealed progressive anemia. A computed tomography (CT) scan showed worsening stenosis of the superior mesenteric vein due to tumor growth and the development of collateral blood vessels around the bile duct–jejunal anastomosis ( Fig. 1 ). He was therefore thought to be experiencing a variceal hemorrhage, owing to tumor-induced extrahepatic portal venous obstruction caused by postoperative recurrence.

**Fig. 1:**
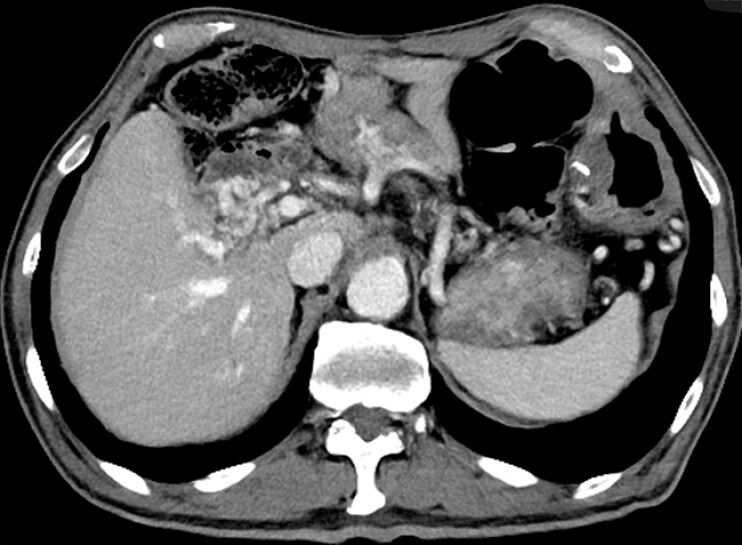
Abdominal computed tomography image showing worsening stenosis of the superior mesenteric vein due to tumor growth and the development of collateral blood vessels around the bile duct–jejunal anastomosis.

When an endoscope (CF H290I; Olympus, Tokyo, Japan) was inserted, a bulge of varices was observed around the bile duct–jejunal anastomosis ( Fig. 2 ). Because we were able to observe them using the endoscope, we chose to perform an EUS-guided vascular intervention. The endoscope was removed and a forward-viewing echoendoscope (TGF-UC260J; Olympus Tokyo, Japan) was reinserted. The forward-viewing echoendoscope, which can be easily advanced into the surgically altered small bowel 5 , enabled us to visualize the varices around the anastomosis ( Fig. 3 ; Video 1 ). We punctured the varices with a 19G needle (EZshot3; Olympus, Tokyo, Japan), and injected a mixture of 1.5 mL Histoacryl and 0.5 mL Lipiodol ( Fig. 4 ). A contrast-enhanced CT scan 3 months after the treatment showed the Histoacryl embolizing the varicose vein ( Fig. 5 ). No complications occurred during treatment. No rebleeding was observed prior to the patient’s death 6 months later from pancreatic cancer.

**Fig. 2:**
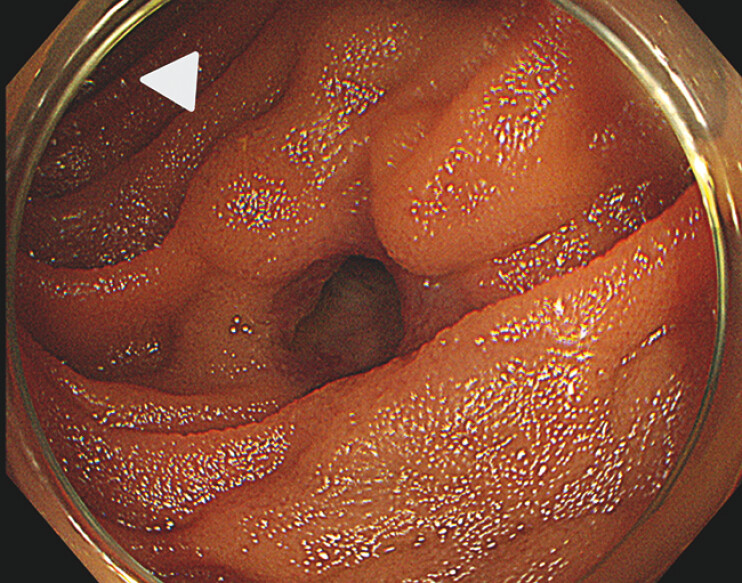
Endoscopic image showing a bulge around the bile duct–jejunal anastomosis (white arrowhead).

**Fig. 3:**
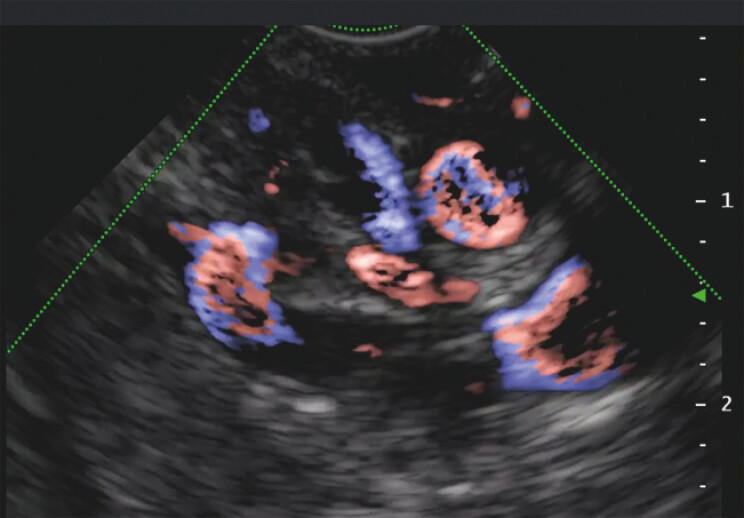
Endoscopic ultrasonography image showing the varicose veins around the anastomosis.

Endoscopic ultrasonography-guided vascular intervention is performed to treat bile duct–jejunal anastomotic variceal bleeding.Video 1

**Fig. 4:**
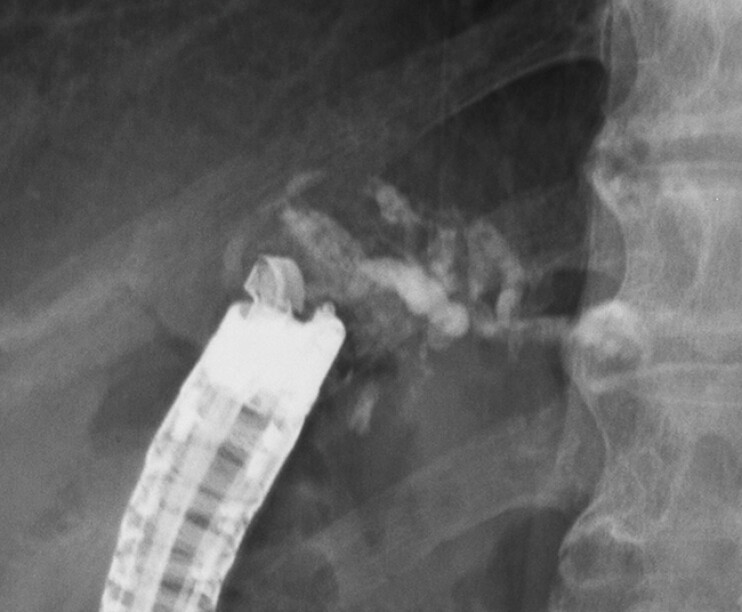
Fluoroscopic image of the injected mixture of Histoacryl and Lipiodol.

**Fig. 5:**
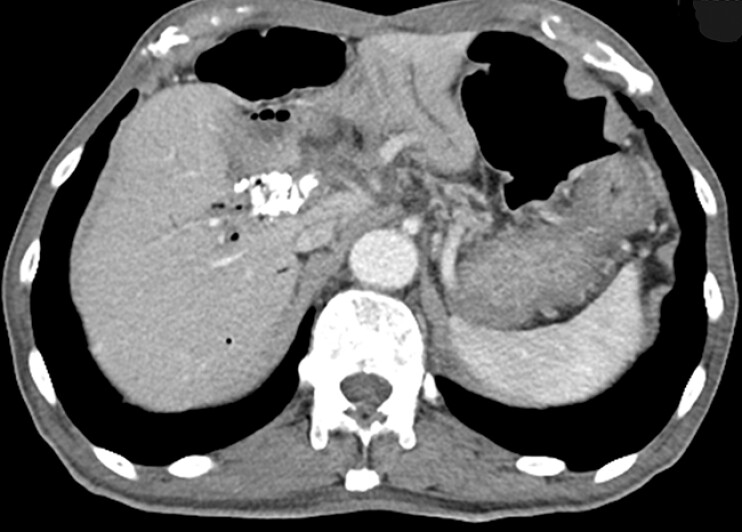
Contrast-enhanced computed tomography scan 3 months after the treatment showing the Histoacryl embolization of the varicose vein.

EUS-guided vascular intervention can be considered an effective treatment for bile duct–jejunal anastomotic varices.

Endoscopy_UCTN_Code_TTT_1AS_2AH

## Notes

Endoscopy E-Videos https://eref.thieme.de/e-videos
*E-Videos* is an open access online section of the journal *Endoscopy* , reporting on interesting cases and new techniques in gastroenterological endoscopy. All papers include a high-quality video and are published with a Creative Commons CC-BY license. Endoscopy E-Videos qualify for HINARI discounts and waivers and eligibility is automatically checked during the submission process. We grant 100% waivers to articles whose corresponding authors are based in Group A countries and 50% waivers to those who are based in Group B countries as classified by Research4Life (see: https://www.research4life.org/access/eligibility/ ). This section has its own submission website at https://mc.manuscriptcentral.com/e-videos .
